# Maternal cardiorespiratory coupling: differences between pregnant and nonpregnant women are further amplified by sleep-stage stratification

**DOI:** 10.1152/japplphysiol.00296.2023

**Published:** 2023-09-28

**Authors:** Maretha Bester, Giulia Perciballi, Pedro Fonseca, Merel M. van Gilst, Massimo Mischi, Judith OEH van Laar, Rik Vullings, Rohan Joshi

**Affiliations:** ^1^Department of Electrical Engineering, Eindhoven University of Technology, Eindhoven, The Netherlands; ^2^Patient Care and Monitoring, Philips Research, Eindhoven, The Netherlands; ^3^Department of Electronics, Information and Bioengineering, Politecnico di Milano, Milano, Italy; ^4^Sleep Medicine Center Kempenhaeghe, Heeze, The Netherlands; ^5^Department of Obstetrics and Gynecology, Máxima Medical Centrum, Veldhoven, The Netherlands

**Keywords:** autonomic regulation, cardiorespiratory coupling, pregnancy, sleep stages

## Abstract

Pregnancy complications are associated with abnormal maternal autonomic regulation. Subsequently, thoroughly understanding maternal autonomic regulation during healthy pregnancy may enable the earlier detection of complications, in turn allowing for the improved management thereof. Under healthy autonomic regulation, reciprocal interactions occur between the cardiac and respiratory systems, i.e., cardiorespiratory coupling (CRC). Here, we investigate, for the first time, the differences in CRC between healthy pregnant and nonpregnant women. We apply two algorithms, namely, synchrograms and bivariate phase-rectified signal averaging, to nighttime recordings of ECG and respiratory signals. We find that CRC is present in both groups. Significantly less (*P* < 0.01) cardiorespiratory synchronization occurs in pregnant women (11% vs. 15% in nonpregnant women). Moreover, there is a smaller response in the heart rate of pregnant women corresponding to respiratory inhalations and exhalations. In addition, we stratified these analyses by sleep stages. As each sleep stage is governed by different autonomic states, this stratification not only amplified some of the differences between groups but also brought out differences that remained hidden when analyzing the full-night recordings. Most notably, the known positive relationship between CRC and deep sleep is less prominent in pregnant women than in their nonpregnant counterparts. The decrease in CRC during healthy pregnancy may be attributable to decreased maternal parasympathetic activity, anatomical changes to the maternal respiratory system, and the increased physiological stress accompanying pregnancy. This work offers novel insight into the physiology of healthy pregnancy and forms part of the base knowledge needed to detect abnormalities in pregnancy.

**NEW & NOTEWORTHY** We compare CRC, i.e., the reciprocal interaction between the cardiac and respiratory systems, between healthy pregnant and nonpregnant women for the first time. Although CRC is present in both groups, CRC is reduced during healthy pregnancy; there is less synchronization between maternal cardiac and respiratory activity and a smaller response in maternal heart rate to respiratory inhalations and exhalations. Stratifying this analysis by sleep stages reveals that differences are most prominent during deep sleep.

## Graphical abstract

**Figure:**
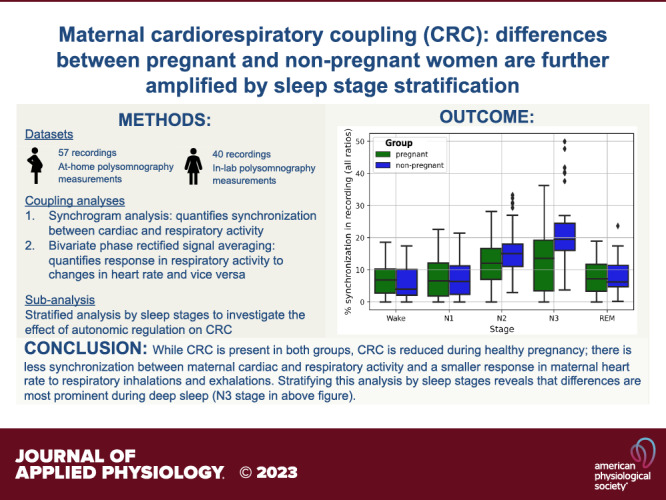

## INTRODUCTION

The maternal autonomic nervous system (ANS) plays an important role in maintaining perinatal health during pregnancy (1). As the ANS is responsible for regulating the function of and interaction between involuntary physiological processes such as heartbeats and respiration, this system is essential in enabling the adaptation of maternal physiology to the growing demands of pregnancy (2). Correspondingly, dysfunctional maternal autonomic regulation has been found in women with pregnancy complications such as hypertensive disorders of pregnancy and gestational diabetes mellitus (3). Pregnancy complications occur in up to 15% of pregnancies and can result in maternal and fetal morbidity and mortality (4–6).

A major hurdle in reducing the impact of pregnancy complications is the inability to detect these complications before the ideal window for medical intervention has passed. Owing to the link between pregnancy complications and autonomic dysfunction, assessing maternal autonomic activity may elucidate subclinical signatures of disease and aid in the early detection of these complications (7). In essence, by identifying when autonomic activity deviates from what is considered normal in a healthy pregnancy, it may be possible to identify high-risk pregnancies earlier (7, 8).

However, while researchers have typically focused on the autonomic dysfunction seen in pregnancy complications (3, 9), the normal autonomic state in a healthy pregnancy is still only partly understood. Noninvasive assessments of autonomic regulation are most often performed by studying heart rate variability (HRV) (10); recent work from our group has shown that there are large, statistically significant differences in a variety of HRV features between healthy pregnant and nonpregnant women (11). However, while HRV is a powerful tool to assess autonomic regulation owing to its relative simplicity and the easy availability of cardiac measurements (10), such assessments capture only a part of the bigger picture of maternal autonomic regulation.

A coherent description of maternal autonomic regulation may be further illuminated by not focusing solely on the heart rate (HR) but rather on the interaction between the cardiac and the respiratory system. This interaction, referred to as cardiorespiratory coupling (CRC), is modulated by the ANS (12) and is present in healthy autonomic states, but weakens or even disappears under diseased or stressed states (13), as well as with aging (14). Correspondingly, the results of two studies indicate altered CRC in pregnant women with preeclampsia (a hypertensive disorder of pregnancy) when compared with healthy pregnant women (15, 16). Yet, surprisingly little is known about CRC in healthy pregnant women and whether this differs from that in nonpregnant women. One study has shown that CRC strength reduces with progressing pregnancy and others have used HRV analyses to show a decrease in high-frequency (HF) cardiac activity (17), which is traditionally linked to the influence of respiration on cardiac activity (10, 18). However, to our knowledge, no previous work has investigated whether there are differences in CRC between pregnant and nonpregnant women.

Subsequently, in the work presented in this paper, we compare CRC between pregnant and nonpregnant women. We do so using two methods one addressing the potential synchronization between the cardiac and respiratory systems (synchrogram analysis), the other addressing a potential modulatory effect between the two systems (bivariate phase-rectified signal averaging analysis). This analysis is performed on data from polysomnography (PSG) studies, which include long periods of synchronized acquisition of ECG and respiratory (thoracic band) signals. Furthermore, as a subanalysis, we stratify the investigation by sleep stages. As CRC is linked to autonomic regulation and each sleep stage is linked to a particular autonomic state (19), this stratification may bring out differences between the groups which may be unobservable if measured over the entire sleep cycle.

## METHODS

In this section, we first detail the retrospective datasets used in the analysis; note that for both datasets, this is a secondary analysis. Next, the preprocessing of both the cardiac and respiratory signals is presented. Furthermore, we describe the two methods that we used to assess CRC. The first method is a phase-locking analysis using the synchrogram method. While there is currently no standard method for assessing CRC, synchrograms are commonly used (20). Second, we use bivariate phase-rectified signal averaging (BPRSA). With this method, the effects of changes in one signal are observed in the other; for example, which activity is observed in the respiratory signal when the HR decelerates. This method, which was developed fairly recently, has previously been used to capture CRC in newborns (21, 22). It has also been used more widely in coupling assessments, for example in assessing baroreflex sensitivity (23–26) as well as capturing the coupling between uterine contractions, and both fetal HR and maternal HR, respectively (27–29). All analyses were performed in Python (PSF).

### Datasets

The pregnancy group comprises healthy volunteers with singleton pregnancies. These women were recruited for an at-home polysomnography (PSG) study, which was conducted in 2015 to validate internal algorithms at Philips Research, Eindhoven, the Netherlands. This volunteer study and its methods, which was carried out in the Netherlands in 2015, were approved by the Internal Committee of Biomedical Experiments of Philips Research, Eindhoven, the Netherlands; all participants provided written informed consent to participate in the study. Participants underwent two full nights of recordings with approximately 8 wk between subsequent sessions, which are denoted as *night 1* and *night 2*. Researchers visited the participants on the eve of recording to set up the Alice PDx, a commercial PSG device that is approved by the US Food and Drug Administration (FDA) for at-home monitoring and consequently adheres to the recommendations for PSG recordings, which recorded ECG at 200 Hz and respiration at 100 Hz using a thoracic band. No abdominal band was attached; this band is only needed for detecting respiratory events such as apneas, which was not the intent of the study. Forty-five women were recruited for this study; of these, 20 had recordings for both nights, 15 had recordings only for *night 1*, and two had recordings only for *night 2*. Eight participants had no recordings available. Of these, two only had one night of attempted recordings while the rest had two nights of attempted recordings. As measurements were performed at home, sensor detachments were common and could not be corrected since the nighttime measurements were not supervised. Note that if an error or detachment occurs for either the ECG or respiration measurement, CRC cannot be determined. Thus, in total, 57 recordings were available. Where two recordings were available per participant, these were viewed as independent owing to the 8-wk gap between the recordings and the substantial physiological changes that occur with advancing gestation (30–32). Participant characteristics are detailed in Table 1.

**Table 1.** Patient characteristics for both groups presented as median and interquartile range

	Pregnant Group	Nonpregnant Group
Age	31 (28–33) yr	24 (21–28)
(Pre-pregnancy) BMI (kg/m^2^)	23.0 (20.7–25.5)	23.1 (22.1–24.6)
Gestational age (all recordings)	24 (20–28) wk	
Gestational age (*night 1*)	21 (18–23) wk	
Gestational age (*night 2*)	28 (26–32) wk	
Average HR	74.6 (68.6–79.1) bpm	62.2 (57.5–67.3) bpm
Average BR	15.5 (14.5–16.8) brpm	15.3 (13.4–16.5) brpm
Time duration of measurement	07:58:12 (07:11:00–08:43:26)	08:38:23 (08:30:12–08:46:56)
Number of measurements	57	40

BMI, body mass index; bpm, beats per minute; BR, breathing rate; brpm, breaths per minute; HR, heart rate.

For the control group, female participants of childbearing age (i.e., 18–45 yr old) were selected from the Healthbed dataset (33). This dataset comprises 110 healthy volunteers who were recruited for a study performed between 2017 and 2018 to collect data for the development of new technologies for sleep assessment. The study was originally approved by the medical ethics committee of Maxima Medical Center, Veldhoven, the Netherlands (W17.128). Participants provided informed consent. The current data analysis protocol (CSG_2022_007) was approved by the medical ethics committee of Sleep Medicine Center Kempenhaeghe, the Netherlands. Pregnancy served as an exclusion criterion for this study. Furthermore, included subjects showed no indications of depression, anxiety, neurological or psychiatric disorders, nor used any medication except birth control. One night of PSG recordings was done per participant at a sleep clinic (Kempenhaeghe, Heeze, the Netherlands), with ECG recorded at 512 Hz and thoracic band respiration at 128 Hz. Characteristics of the 40 women who met the criteria for this analysis are detailed in Table 1. All research was conducted in accordance with the Declaration of Helsinki.

### Signal Preprocessing and Fiducial Point Detection

Data for the pregnant group were recorded at home, i.e., in free-living conditions. Subsequently, there were several regions with sensor detachment and clear motion artifacts. These were removed based on visual inspection during exploratory data analysis. Thereafter, for both groups, a second-order notch filter was used to remove the 50-Hz powerline interference for both ECG and respiration. Respiration was further band-pass filtered between 0.05 and 0.6 Hz (corresponding to 3 to 36 breaths per minute) to remove noise and facilitate peak and trough detection. Respiratory peaks and troughs were detected using a published algorithm (34) from NeuroKit2, a toolbox designed for neurophysiological signal processing in Python (35). Furthermore, the respiratory signals were normalized by subtracting the mean value of the signal from itself and thereafter dividing the signal by the median of its absolute values (21). ECG R-peaks were also detected using a published peak detector (36) and, subsequently, the corresponding tachograms were calculated. RR intervals were rejected if they fell outside the range of 0.5–2 s (corresponding to 30 to 120 heartbeats per minute) or differed from the preceding interval by more than 20%.

### CRC Assessment with Synchrograms

Synchrograms are used to visualize and detect periods where a fixed relative phase relationship exists between two oscillatory signals, such as the ECG and respiratory signals. This type of CRC is referred to as cardiorespiratory phase synchronization (CRPS). We provide a brief, contextualized overview of this method here. For further details, please see the original introduction of the method (37), as well as additional articles providing detailed examples using synchrograms (38, 39).

The synchrogram provides a stroboscopic view of the phase of the respiration signal at the times of the R-peaks of the ECG waveform. As an example, assume that four (*n* = 4) R-peaks occur within one respiration cycle (*m* = 1). Each of these R-peaks would then occur at a certain phase relative to the corresponding respiratory cycle. If in the successive respiratory cycles, sets of four R-peaks again occur at the same fixed phases relative, within a threshold defined based on the literature, to their corresponding respiratory cycles, then phase locking is present at a ratio of 4:1 (*n:m*) for that period. This phase lock needs to be present for a minimum of two cycles of *m* (in this example, for two respiratory cycles) for CRPS to occur.

The fixed integer relationship of *n:m* is referred to as the phase locking ratio (PLR). We investigate the following PLRs: 3:1, 4:1, 5:1, 6:1, 7:1, 5:2, 7:2, 9:2, and 11:2, expecting based on literature that 3:1, 4:1, 5:1, and 6:1 would occur most frequently (19, 40, 41). Furthermore, we also assess all ratios, i.e., the sum of the CPRS detected for each of the specified ratios. CRPS is calculated across the entirety of the signals and results are presented as the percentage of time in which CRPS occurs, to ensure the results are independent of recording length and sleep architecture; the latter is elaborated upon in *Subanalysis: Stratification by Sleep Stages*.

**Figure 1.**
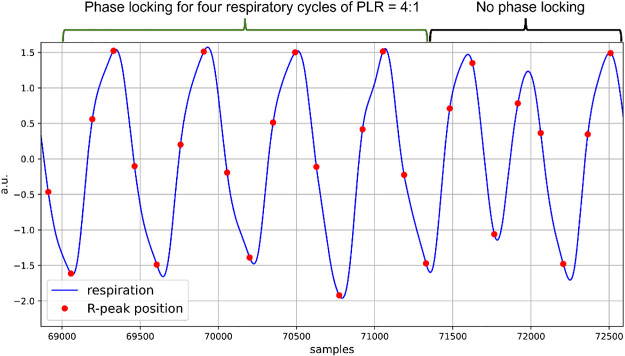
Illustration of a period of CRPS, with instances of the R-peaks plotted in red on the respiratory signals. Notice that for the first four respiratory cycles, phase locking of PLR 4:1 occurs. This is evident from the fact that the R-peaks (in red) occur at similar fixed positions relative to the respiratory cycles. Thereafter, no phase locking occurs. CRPS, cardiorespiratory phase synchronization.

To further illustrate CRPS, an example from one of the study participants is presented in Fig. 1. Notice that each of the first four respiratory cycles has four red points, which correspond to the occurrence of the R-peak from the time-synchronized ECG signal, at similar relative positions. This pattern breaks down for the subsequent three respiratory cycles. Therefore, for the first four respiratory cycles, a phase locking of a 4:1 PLR occurs, while no phase locking occurs thereafter.

### CRC Assessment with BPRSA

BPRSA is the bivariate version of phase-rectified signal averaging (PRSA). The latter is a technique that is used to elucidate quasiperiodicities in physiological signals that may otherwise be obscured by noise. This method, as well as its bivariate version discussed in the next paragraph, is presented in detail elsewhere (42–44); here, we give a brief overview pertaining to the analyses presented in this paper. Note that when using BRPSA to capture coupling, the PRSA and BPRSA results are typically both presented to better illustrate the coupling between the two signals. Hence, we describe both the PRSA and BPRSA analysis here (21, 27, 28, 42, 45).

The PRSA analysis is performed by first identifying the events or phases of interest, referred to as anchor points (APs), in the relevant signal. Following this, a signal segment of length 2 *L* + 1 is isolated around each AP, with *L* empirically chosen to allow for visualizing the slowest oscillation of interest. Next, all signal segments are aligned by their APs and averaged to provide the resulting waveform. This averaging ensures that only periodicities that have a fixed relationship with the AP remain. If no such relationship exists, the PRSA waveform will be a flat line. Alternatively, we can observe the typical behavior of the signal around each AP in the waveform. Normally, this is observed as an oscillation around the AP which has a certain period of coherence; outside of this period of coherence, the oscillations taper off.

An extension of this method allows for studying the coupling between two signals. BPRSA, i.e., the bivariate version of PRSA, captures if and how specific events or phases in one signal (the trigger signal) might correspond to or result in changes in another signal (the target signal). This is done by identifying the APs in the trigger signal and translating them in time to the target signal. From here, the rest of the analysis, as specified for PRSA, is performed on the target signal. If the resulting BPRSA waveform resembles a flat line, no coupling is present (as assessed by this specific method). However, if periodicities are present in the BPRSA waveform, this indicates that some activity in the target signal corresponding to the AP event in the trigger signal survives the averaging, in which case coupling is observed. In this case, similar to that of the PRSA waveform described in the previous paragraph, an oscillation is expected around the AP with a certain period of coherence, and outside this period, the oscillations in the waveform will taper off. Specifically, in the terminology used for BPRSA analysis, the coupling is observed from the trigger signal to the target signal. While we use this language throughout, it should be noted that causality is not necessarily implied. Note that the PRSA and BPRSA analysis is performed using the full signals.

#### PRSA of tachogram and respiration.

We identify HR accelerations and HR decelerations as the two sets of APs for the tachogram, meaning that each HR deceleration and HR acceleration is identified as an AP, respectively. An HR acceleration is identified when an RR interval is shorter (faster) than its preceding RR interval and, correspondingly, an HR deceleration is identified when an RR interval is longer (slower) than its predecessor. The time point of the AP is denoted as the end of the accelerated or decelerated cardiac cycle, i.e., the second R-peak in the RR interval within the acceleration/deceleration segment. For the respiratory signal, inhalations and exhalations serve as APs. Note that for inhalation we use the point halfway in terms of time between the trough and the peak of the respiratory cycle; similarly, for exhalation, we use the halfway point between the peak and the trough. This halfway point was preferred, as the inhalation peak and exhalation trough already represent the transition from inhalation to exhalation and exhalation to inhalation, respectively.

The following features are calculated to characterize the PRSA waveform of the tachogram, using HR decelerations as APs. Note that for the PRSA of the tachogram, the waveform’s relationship to the time domain is units of RR values (specified here as RRi) and not seconds:

•  Deceleration capacity (DC) (44): this feature captures the response in HR to decelerations and is calculated in the following way:
C=[X(0)+ X(1)− X(−1)− X(−2)]/4,

with *X* representing the PRSA waveform, *X*(0) representing the AP, *X*(1) is the value following the AP, while *X*(−1) and *X*(−2) precede the AP.

•  Immediate deceleration response (IDR) (21): the difference between the maximum and minimum value within the neighborhood of five RRi preceding the AP and five thereafter, including the AP.
•  Slope of the deceleration response (SDR) (21): the slope between the maximum and minimum value as defined for the IDR.

These features are also calculated in the case where HR accelerations are the APs and correspondingly named: acceleration capacity (AC), immediate acceleration response (IAR), and slope of the acceleration response (SAR). As these features capture the beat-to-beat information of the tachogram, they mainly reflect parasympathetic activity.

The PRSA waveform of the respiratory signal, both when inhalations and exhalations are APs, is characterized using the following features:

•  Maximum respiratory amplitude (MRA) (21): the difference between the maximum and minimum values of the PRSA within 5 s preceding or following the AP. This feature captures the maximum response to the AP.
•  Sample entropy (SampEn) (21): the sample entropy is calculated for the PRSA waveform. A small value corresponds to higher regularity, whereas a higher value implies more randomness in the oscillations of the waveform. The tolerance was set to 0.2 times the standard deviation of the waveform, while the embedding dimension was set to 4.

#### BPRSA of the tachogram and respiratory signal from cardiac activity to respiration.

To quantify CRC from cardiac activity to respiration, we perform the BPRSA analysis with the tachogram as the trigger signal and the respiratory signal as the target signal. We use HR decelerations and HR accelerations as APs, respectively. Conversely, we use respiration as the trigger signal and the tachogram as the target signal to quantify CRC from respiration to cardiac activity.

We quantify the resulting BPRSA waveforms with SampEn as described in the previous section (*PRSA of tachogram and respiration*). Furthermore, we calculate the maximum BPRSA amplitude (MBA) in the same manner as MRA is calculated (*PRSA of tachogram and respiration*). In addition, we also calculate the slope at the AP (SAP) (21) to identify the phase of tachogram or respiration at the AP (a negative value corresponds to HR acceleration or the expiratory phase, respectively, whereas a positive value corresponds to HR deceleration or the inspiratory phase, respectively). Finally, we also calculate the peak delay (PD), i.e., the delay between the peak/trough of the BPRSA waveform aligning with the AP and the *x*-axis, essentially capturing the time offset between the AP and the peak/trough.

### Subanalysis: Stratification by Sleep Stages

As the data from both groups were recorded during the night and sleep stages are characterized by different autonomic tones (46), we stratify our analysis by sleep stage. Sleep can be characterized in three states of increasing depth (N1, N2, and N3), as well as rapid eye movement (REM). Sleep scoring is done on both datasets using a PSG-based automated sleep stager, Somnolyzer 4.0 (an optional component of the Sleepware G3 software; Philips Respironics, Pittsburgh, PA), which is detailed elsewhere (47, 48). For the pregnant group, 10 recordings did not have the relevant signals to determine the sleep stages and were not included here, resulting in the inclusion of 47 recordings.

To illustrate how this subanalysis is performed, the N2 sleep stage is used as an example. All segments of N2 sleep are isolated from the recording. Segments shorter than 1 min are disregarded for the synchrogram analysis and those shorter than 3 min are discarded for the BPRSA analysis, based on the minimum length needed for these methods (note that sleep staging is done in 30 s of nonoverlapping increments). The synchrogram analysis, detailed in *CRC Assessment with Synchrograms*, is performed on each N2 segment. The results from all segments are weighted according to the length of the relevant segment as a fraction of the total length of the PSG recording spent in N2. Concerning the BPRSA analysis (*CRC Assessment with BPRSA*), only data from the N2 sleep stage are used for the analysis. Apart from this change, the analysis remains identical. The same process is repeated for all sleep stages, as well as for the sections where participants were identified as awake (Wake = W).

### Subanalysis: Investigating the Effect of Gestational Age

Using both methods for CRC assessment, we perform an additional subanalysis in the pregnant group to explore the effects of gestational age (i.e., the number of weeks participants had been pregnant) on CRC. We investigate whether any of the CRC indices, from both the synchrogram of BPRSA analysis, differs with gestational age. We perform two investigations. First, we do a pairwise comparison between the CRC indices of women who had two nights of measurements approximately 8 wk apart. Second, as discussed in *Datasets*, we treat each recording as independent and perform a simple regression analysis with gestational age as the independent variable and each CRC index as the dependent variable, respectively i.e., an individual regression analysis is performed for each CRC index.

### Subanalysis: Investigating the Effect of Age

Similarly, we investigate whether there is a trend in the CRC parameters relative to age. To this end, we again perform a linear regression using age as the independent variable and CRC indices as dependent variables. Specifically, we investigate all features describing the BPRSA waveforms (as laid out in the section *BPRSA of the tachogram and respiratory signal from cardiac activity to respiration*) and the total CRPS for each participant. We use all results from the nonpregnant group and the results of the first night of measurements for the pregnant group.

### Statistical Analysis

Almost all data and results were non-normally distributed and, subsequently, nonparametric analyses were performed. Statistical significance for changes between the pregnant and nonpregnant group was tested using the Mann–Whitney *U* test. When comparing differences between the sleep stages (i.e., differences between more than two groups), the Kruskal–Wallis test was used. In addition, Cohen’s *d* was calculated to determine effect size. The effect size is presented with the 95% confidence interval, obtained via bootstrapping with 10,000 iterations, which is appropriate for data that is not normally distributed (49). A *d*-value of 0.8 suggests a large effect size, while *d* = 0.5 and *d* = 0.3 correspond to a medium and small effect size, respectively.

## RESULTS

The results for the synchrogram and BRPSA analyses are presented in this section, first when calculated over the full recordings, followed by the findings from the subanalysis in which the analyses are stratified by sleep stages. Note that when investigating the effect of gestational age on CRC (the subanalysis described in *Subanalysis: Investigating the Effect of Gestational Age*), we found no significant or remarkable results. Similarly, when examining the effect of age on CRC (described in *Subanalysis: Investigating the Effect of Age*), we found no statistically significant relationships. As such, these results are not further presented here.

**Figure 2.**
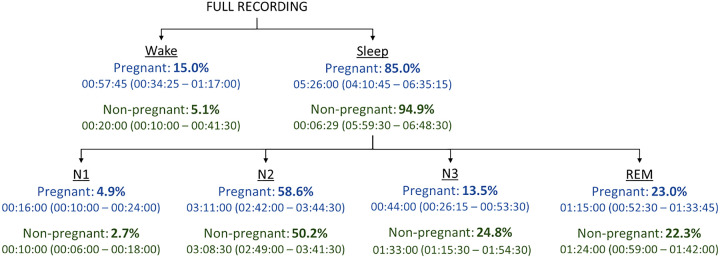
Data were recorded during the nighttime. The split of these recordings between Wake and Sleep is presented on the first tier, adding up to 100% per group. In the second tier, the time spent in Sleep is broken up into the four sleep stages (N1, N2, N3, and REM). Again, these proportions add up to 100% per group. The proportions presented here are calculated based on the median times spent in each stage per group. The median and interquartile range of the times spent in each stage are presented as hours: minutes: seconds. REM, rapid eye movement.

Concerning the sleep-stage stratification, we broke down the full recordings into the different stages to contextualize the results relative to the time spent in each stage (Fig. 2). The values displayed in Fig. 2 are based on the synchrogram analysis, for which sleep stage segments shorter than 1 min were discarded. Note that for the BPRSA analysis, these values differ slightly as segments of at least 3 min in length are needed. Still, the proportional time spent in each stage remains similar.

For pregnant women, a larger proportion of the recording time is spent in Wake (15.0% vs. 5.1% in non-pregnant women); this agrees with the literature as pregnant women are known to have more wakeful periods than nonpregnant women (50, 51). Furthermore, considering the time spent in sleep, both groups spent most of their sleep in N2 (>50%) and little time in N1 (<5%). Both groups also spent comparable time in REM sleep (23.0% and 22.3%). However, the proportion of time pregnant women spent in N3 sleep is remarkably less than that of nonpregnant women (13.5% vs. 24.8%), which is expected from the literature (50, 51). Further aligning with the literature, pregnant women then spent almost 10% more of their sleep in N2 than their nonpregnant counterparts (51).

### CRC Assessment with Synchrograms

**Figure 3.**
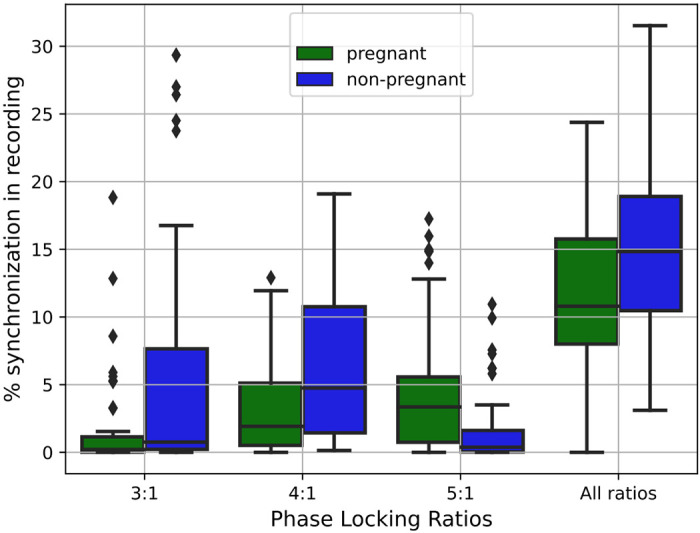
The percentage of synchronization for the pregnant (in green) and nonpregnant (in blue) group, presented for the most commonly occurring PLR (i.e., 3:1, 4:1, and 5:1), as well as for all ratios combined. For pregnant women, less synchronization occurs at 3:1, 4:1, and all ratios, while these women have more synchronization for 5:1 when compared with nonpregnant women. The results presented in this figure are further outlined in Table 2 along with their statistical significance and effect sizes. PLR, phase locking ratio.

First, the synchrogram analysis was performed on the full recordings. Figure 3 visualizes the results for all the PLRs (i.e., phase-locking ratios) combined, as well as 3:1, 4:1, and 5:1, the most commonly occurring PLRs, with boxplots. The median and interquartile of the percentage of time spent in each PLR, along with the statistical significance (*P*) and effect sizes (*d*) of differences between the groups, are found in Table 2.

**Table 2.** Differences in the percentage of CRPS between pregnant and nonpregnant women, broken down by the different ratios

Ratios	Pregnant	Nonpregnant	Significance	Effect Size
All	10.79 (8.00–15.76) %	14.84 (10.47–18.90) %	*P* < 0.01	*d* = 0.60 (0.20–1.00)
3:1	0.21 (0.02–1.06) %	0.76 (0.22–7.65) %	*P* < 0.01	*d* = 0.62 (0.24–0.98)
4:1	1.88 (0.15–5.03) %	4.75 (1.44–10.75) %	*P* < 0.01	*d* = 0.66 (0.24–1.10)
5:1	3.26 (0.38–5.45) %	0.38 (0.03–1.62) %	*P* < 0.01	*d* = 0.64 (0.28–0.99)
6:1	0.20 (0.00–1.43) %	0.00 (0.00–0.07) %	*P* < 0.001	*d* = 0.65 (0.46–0.85)
7:1	0.00 (0.00–0.03) %	0.00 %	*P* = 0.01	*d* = 0.36 (0.01–0.63)
5:2	0.00 %	0.00 (0.00–0.04) %	*P* < 0.001	*d* = 0.48 (0.10–0.80)
7:2	0.39 (0.00–1.65) %	0.00 (0.04–0.34) %	*P* < 0.01	*d* = 0.35 (−0.06–0.78)
9:2	0.13 (0.00–0.44) %	0.39 (0.18–0.45) %	*P* = 0.47	*d* = 0.05 (−0.37–0.46)
11:2	0.06 (0.04–0.34) %	0.00 %	*P* = 0.01	*d* = 0.55 (0.26–0.81)

CRPS values are presented as median and interquartile range. CRPS, cardiorespiratory phase synchronization.

Overall, significantly less CRPS occurs during pregnancy (Fig. 3, all ratios). In nonpregnant women, a median of 15% of the night is spent in CRPS, whereas in pregnant women, CRPS occurs only a median of 11% of the time. Furthermore, significantly less CRPS of the PLRs 3:1 and 4:1 occur during pregnancy. However, there is a higher prevalence of the PLR of 5:1 in the pregnant group. In all cases *d* ≈ 0.6, which corresponds to a medium effect size.

When considering the additional PLRs such as 7:1 or 9:2 (Table 2), we see that these PLRs rarely occur in either of the groups. Still, there are mostly statistically significant differences between the groups, regardless of the PLR. Furthermore, PLRs with a higher number of heartbeats per respiration cycle are more likely to occur in the pregnant group.

#### Stratification by sleep stages.

**Figure 4.**
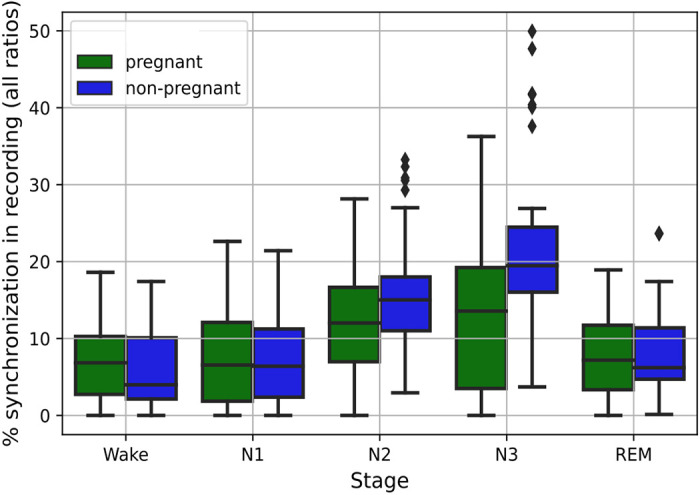
Synchronization periods stratified by sleep stages. The percentage of synchronization for the pregnant (in green) and nonpregnant (in blue) group, stratified by sleep stages. The results presented here are for all PLRs combined. Comparable synchronization occurs during Wake, N1, and REM, but pregnant women have reduced synchronization in N2 and N3 compared with nonpregnant women. N1, N2, and N3 are sleep stages; PLR, phase locking ratio; REM, rapid eye movement.

To investigate whether differences in autonomic regulation between the groups are driving their differences in CRC, the synchrogram analysis was stratified by the four sleep stages (N1, N2, N3, and REM) and Wake. The proportion of time each group spends in the different stages was previously outlined in Fig. 2. In Fig. 4, the percentage of time spent on CRPS (combining all PLRs) is presented as stratified by sleep stages. The least amount of CRPS in sleep occurs during N1 and REM sleep (a median of ∼5–7% for both groups in both stages), while the most occur during N3 (a median of 13% and 20% for pregnant and nonpregnant women, respectively). Pregnant women have more CRPS during Wake, but it should be kept in mind that a larger proportion of Wake measurements are available for this group (Fig. 2). When comparing overall CRPS between the different sleep stages, this differs significantly for both pregnant (*P* = 0.0001) and nonpregnant women (*P* < 0.0001).

Furthermore, there are significant (*P* < 0.05) and medium effect size differences (*d* = 0.62) between the groups during N2, as well as large differences between the groups during N3 (*P* < 0.001, *d* = 0.93), whereas for N1 and REM, the differences between groups are less evident and not significant. As sleep deepens, quantified by the progression from N1 to N3, the differences between the groups become more pronounced. For nonpregnant women, there is a delta of ∼15% between the medium CRPS in N1 and N3, whereas for pregnant women, this difference is only ∼8%.

### CRC Assessment with BPRSA

**Figure 5.**
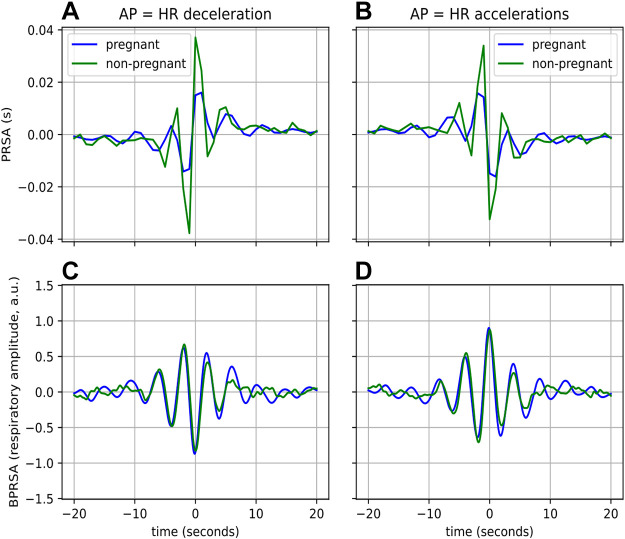
Coupling from cardiac activity to respiration, averaged across all participants. In the *A* and *C*, AP = HR deceleration, and in *B* and *D*, AP = HR accelerations. The top row represents the result from the PRSA (*A* and *B*), while the bottom row is that of BPRSA (*C* and *D*). The means of the waveforms have been subtracted to facilitate comparison. AP, anchor point; BPRSA, bivariate phase-rectified signal averaging; HR, heart rate; PRSA, phase-rectified signal averaging.

**Figure 6.**
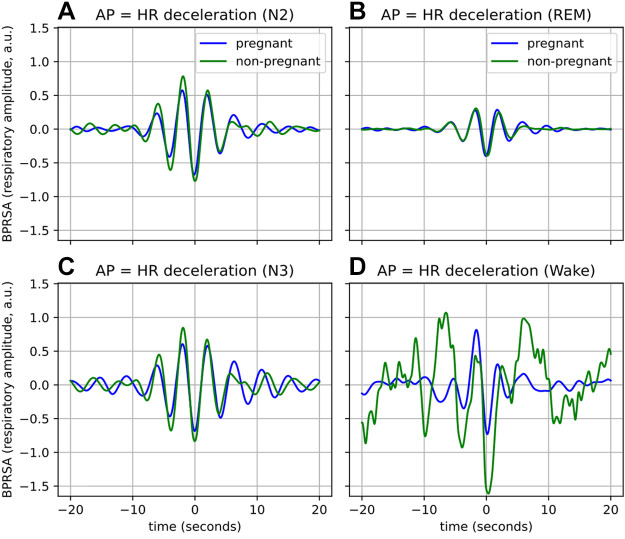
The average BPRSA waveforms representing coupling from cardiac activity to respiration during the N2 (*A*), N3 (*C*), REM (*B*), and Wake (*D*) for AP = HR decelerations. The means of the waveforms have been subtracted to facilitate comparison. Waveforms are averaged across all participants. AP, anchor point; BPRSA, bivariate phase-rectified signal averaging; HR, heart rate; PRSA, phase-rectified signal averaging; REM, rapid eye movement.

**Figure 7.**
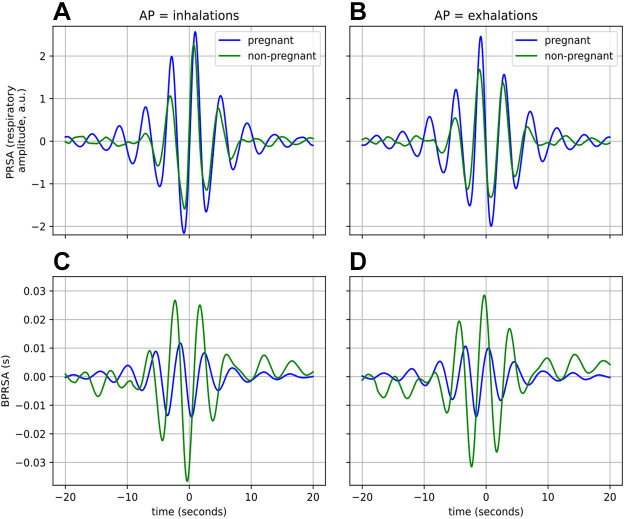
Coupling from respiration to cardiac activity. In *A* and *C*, AP = inhalations, and in *B* and *D*, AP = exhalations. The top row represents the result from the PRSA (*A* and *B*), while the bottom row is that of BPRSA (*C* and *D*). The means of the waveforms have been subtracted to facilitate comparison. Waveforms are averaged across all participants. AP, anchor point; BPRSA, bivariate phase-rectified signal averaging; PRSA, phase-rectified signal averaging.

**Figure 8.**
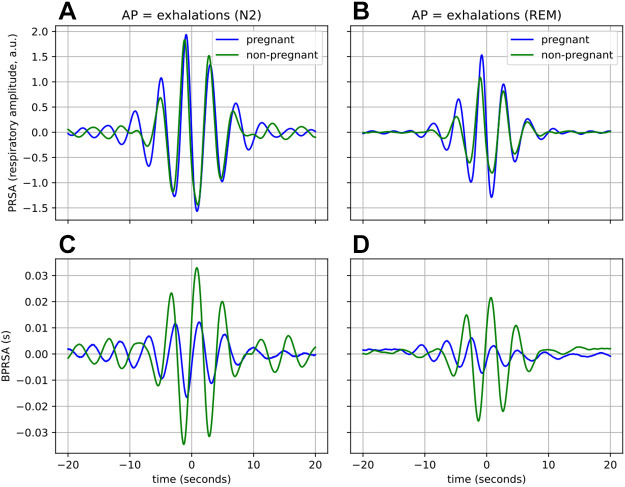
Coupling from respiration to cardiac activity during the N2 (*left*, *A* and *C*) and REM sleep stage (*right*, *B* and *D*), with AP = exhalations. The top row represents the result from the PRSA (*A* and *B*), while the bottom row is that of BPRSA (*C* and *D*). The means of the waveforms have been subtracted to facilitate comparison. Waveforms are averaged across all participants. AP, anchor point; BPRSA, bivariate phase-rectified signal averaging; PRSA, phase-rectified signal averaging; REM, rapid eye movement.

The results from the BPRSA analysis are presented in this section, first performed using the entire signal and thereafter stratified by sleep stages. There are two overarching observations to notice from these results. First, from Figs. 5, 6, 7, and 8, it is evident that there is a relationship between the cardiac and respiratory systems for both pregnant and nonpregnant women, regardless of whether HR accelerations, HR decelerations, inhalations, or exhalations are used as APs. Recalling *CRC Assessment with BPRSA*, CRC is present if the resulting BPRSA waveform contains oscillations, i.e., not a flat line. The second observation is that while CRC is present in both groups, the nature of the CRC differs between pregnant and nonpregnant women.

#### Coupling from cardiac activity to respiration.

We perform the BPRSA analysis with HR decelerations and accelerations as APs, respectively. The PRSA and BPRSA plots of the pregnant and nonpregnant groups, averaged per group, are presented in Fig. 5. The mean values of the waveforms in this figure, as well as those in Figs. 6, 7, and 8, have been subtracted to facilitate comparison between the groups. From the PRSA waveforms in this figure (Fig. 5, *A* and *B*), we can clearly observe a response for both groups, which is evidenced by the prominent oscillations observed around the APs. Note that the APs correspond to the midpoint of the *x*-axis (time = 0 s). These oscillations taper off to both sides after ∼10 s. We see a substantially larger response for nonpregnant women than for pregnant ones in both cases of APs. This is confirmed by the statistically significant (*P* < 0.001) and large (*d* > 1.15) differences in the features in Table 3, which capture the response observed in the PRSA waveforms (DC, AC, IDR, IAR, SDR, and SAR).

**Table 3.** Features describing the PRSA and BPRSA waveforms when CRC is assessed from cardiac activity to respiration

	Feature	Pregnant	Nonpregnant	*P*	Effect Size (*d*)
*AP = HR deceleration*					
PRSA
	IDR	0.035 (0.026–0.044)	0.067 (0.034–0.102)	<0.001	1.28 (0.92–1.67)
	SDR (× 10^3^)	12.1 (0.76 to –1.79)	5.82 (2.75–8.71)	<0.001	1.42 (1.06–1.84)
	DC (× 10^2^)	1.49 (1.14–1.81)	2.68 (1.48–3.67)	<0.001	1.20 (0.87–1.57)
BPRSA	MBA	1.37 (1.07–1.53)	1.55 (1.45–1.76)	<0.001	0.25 (−0.25–1.07)
	PD, ms	−11 (−19–0.5)	4 (−13–28)	0.03	0.39 (−0.04–0.84)
	SAP (× 10^3^)	2.10 (0.00–3.91)	−0.31 (–2.96–1.33)	<0.01	0.46 (−0.15–0.85)
	SampEn (× 10^2^)	4.53 (3.99–4.84)	3.62 (3.08–4.08)	<0.001	0.75 (0.26–1.33)
*AP = HR acceleration*					
PRSA	IAR	0.035 (0.027–0.046)	0.062 (0.039–0.094)	<0.001	1.27 (0.86–1.7)
	SAR (× 10^3^)	−12.2 (−18.5 to −7.3)	−49.1 (−80.8 to −27.9)	<0.001	1.43 (1.02–1.88)
	AC (× 10^2^)	−1.50 (−1.87 to −1.21)	−2.64 (−3.24 to −1.61)	<0.001	1.16 (0.75–1.6)
BPRSA	MBA	1.46 (1.30–1.67)	1.67 (1.48–1.85)	<0.01	0.22 (−0.25–0.93)
	PD, ms	−11 (−18.5 to −1)	5 (−12–18)	<0.01	0.41 (0.00–0.85)
	SAP (× 10^3^)	−2.40 (−4.37 to −0.25)	0.51 (−1.94–2.75)	<0.01	0.56 (0.28–1.01)
	SampEn (× 10^2^)	4.49 (4.0–4.85)	3.52 (3.07–4.07)	<0.001	0.75 (0.26–1.32)

Features are presented as median and interquartile range. AP, anchor point; BPRSA, bivariate phase-rectified signal averaging; CRC, cardiorespiratory coupling; HR, heart rate; MBA, maximum BPRSA amplitude; PD, peak delay; PRSA, phase-rectified signal averaging; SAP, slope at the anchor point.

By observing the BPRSA waveforms (Fig. 5, *C* and *D*), we can see that coupling clearly exists between the trigger and target signals. This is evident from the prominent oscillations (i.e., not a flatline) observed around the APs at the midpoint of the *x*-axis, which taper off to both sides after ∼10 s. Now consider the PRSA waveforms alongside the the BPRSA waveforms; notice that the HR decelerations are clustered at the end of the expiratory phase, while HR accelerations cluster at the end of the inspiratory phase. Based on visual observation, the BPRSA waveforms of the two groups are similar (Fig. 5, *C* and *D*). However, based on the statistical analysis of the features describing the BPRSA waveforms in Table 3, there are statistically significant differences between the BPRSA waveforms of the two groups, albeit with generally small to medium effects.

#### Stratification by sleep stages: coupling from cardiac activity to respiration.

To delve further into the potential physiological drivers behind the differences in the groups, the BPRSA analysis from *Coupling from cardiac activity to respiration* was stratified by sleep stages. The PRSA graphs remained similar across sleep stages, with nonpregnant women always showing a larger response than their pregnant counterparts (results not shown). However, the BPRSA waveforms, which indicate the response in respiration corresponding to changes in HR, did differ per sleep stage. The average waveforms are presented in Fig. 6. These waveforms are for the case of AP = HR decelerations; the BPRSA results of AP = HR accelerations were similar. The average waveforms for the N1 sleep stage are not presented here, as too little N1 data were available (Fig. 2).

Notice that for N2 and N3 (Fig. 6, *A* and *C*), the difference in the maximum response (MBA), which is not visually obvious in Fig. 5*C*, becomes apparent. To further illustrate this difference, in N2, the effect size of the difference is large (*d* = 0.88), whereas the effect size of the differences based on the full recordings is small (*d* = 0.25, Table 3). However, the difference between the groups disappears when looking at data from the REM stage (Fig. 6*B*). Furthermore, the amplitudes of both the BPRSA waveforms in the REM phase are reduced compared with that of N2 and N3, as well as to the BPRSA waveform based on the full recording (Fig. 5*C*). Note that for the Wake results of the nonpregnant group (Fig. 6*D*), the jagged appearance of the waveform likely results from the little Wake data available for this group (Fig. 2) rather than physiological differences between the two groups.

#### Coupling from respiration to cardiac activity.

In addition, we performed the BPRSA analysis using inhalations and exhalations as APs, respectively. The average waveforms for both groups are presented in Fig. 7, with the corresponding descriptive features found in Table 4. We see from the PRSA waveforms that there is a larger response for the pregnant group, although this difference is only significant (*P* < 0.01) for the case where exhalations are APs (see MRA for Table 4). However, looking at the BPRSA waveforms and features in Table 4, there is a clear difference in the amplitudes of the responses, with the larger response belonging to the nonpregnant group. These differences are echoed in the features in Table 4 for both inhalations and exhalations as APs: there are statistically significant (*P* < 0.001) and large (*d* > 1.2) differences in MBA, i.e., the amplitude of the response observed in the waveform. Furthermore, for both the PRSA and BPRSA waveforms, considering both sets of APs, there is significantly lower SampEn in the response of the pregnant women (*P* < 0.001, *d* > 1).

**Table 4.** Features describing the PRSA and BPRSA waveforms when CRC is assessed from respiration to cardiac activity

	Feature	Pregnant	Nonpregnant	*P*	Effect Size (*d*)
*AP = INHALATION*					
PRSA	MRA	3.67 (3.41–4.12)	3.61 (3.34–3.77)	0.18	0.06 (−0.39–0.36)
	SampEn (× 10^2^)	4.35 (3.98–4.84)	3.40 (2.92–3.71)	<0.001	1.16 (0.71–1.70)
BPRSA	MBA	0.036 (0.018–0.046)	0.068 (0.045–0.091)	<0.001	1.28 (0.96–1.63)
	PD, ms	36 (2–70)	12 (−47–58.25)	0.02	0.52 (0.09 – 0.95)
	SAP (× 10^3^)	−0.108 (−0.304–0.000)	−0.069 (−0.243–0.105)	0.03	0.48 (0.07–0.88)
	SampEn (× 10^2^)	4.69 (4.25–5.14)	3.26 (2.98–3.88)	<0.001	1.48 (1.09–1.99)
*AP = EXHALATION*					
PRSA	MRA	3.37 (3.19–3.87)	3.19 (3.04–3.36)	<0.01	0.19 (−0.23–0.62)
	SampEn (× 10^2^)	4.48 (3.98–4.88)	3.46 (3.07–3.98)	<0.001	1.08 (0.62–1.64)
BPRSA	MBA	0.034 (0.017–0.043)	0.066 (0.044–0.090)	<0.001	1.35 (1.02–1.71)
	PD, ms	42 (−11–79)	29 (−33.75–72.25)	0.20	0.23 (−0.2–0.65)
	SAP (× 10^3^)	−0.056 (−0.025–0.180)	0.077 (−0.055–0.180)	0.44	0.29 (−0.15–0.68)
	SampEn (× 10^2^)	4.76 (4.43–5.30)	3.31 (3.06–3.90)	<0.001	1.31 (0.82–2.17)

Features are presented as median and interquartile range. AP, anchor point; BPRSA, bivariate phase-rectified signal averaging; CRC, cardiorespiratory coupling; MBA, maximum BPRSA amplitude; PD, peak delay; PRSA, phase-rectified signal averaging; SAP, slope at the anchor point.

#### Stratification by sleep stages: coupling from respiration to cardiac activity.

Next, the BPRSA analysis using respiration as the trigger signal and the tachogram as the target signal was stratified by sleep stages. The left panel of Fig. 8 shows the average PRSA and BPRSA waveforms from the N2 sleep stage for AP = exhalations, which is when parasympathetic activity is dominant. The right panel shows that of the REM sleep stage, where sympathetic activity is dominant (19). The results of AP = inhalations are not shown as these are similar to those presented in Fig. 8.

In Fig. 7, *B* and *D*, where the waveforms are based on the full night recordings, we can observe a difference in the amplitude of the PRSA waveforms with a small effect size (MRA from Table 4, *P* < 0.01, *d* = 0.19). From Fig. 8, it seems that this difference is primarily driven by the large, significant differences in sympathetic states such as the REM stage (*P* < 0.001, *d* = 1.61). Considering now the BPRSA waveforms, we see that there is a substantially larger response for nonpregnant women, as compared with pregnant ones, for both N2 and REM. This is similar to the results seen in Fig. 7*D* and this is also the case for N3 and Wake (not shown).

Furthermore, considering the values for SampEn for both the PRSA and the BPRSA waveforms for both sets of APs, there is significantly less regularity in the waveforms of the pregnant group, with a large effect (*d* > 1, *P* < 0.001), regardless of the sleep stage. This indicates that there is a higher level of randomness in the oscillations of the waveforms of the pregnant group, while the waveforms of nonpregnant women show more regularity.

## DISCUSSION

Although it is known that pregnancy substantially impacts the cardiac, respiratory, and autonomic nervous systems of women, we show for the first time in this work that pregnant women have altered CRC when compared with their nonpregnant counterparts. Using two different CRC analyses, we find that the synchronization between the cardiac and respiratory systems (CRPS in Fig. 2), as well as the effect of respiration on cardiac activity (Fig. 7), are reduced during a healthy pregnancy. We further stratify these analyses by sleep stages. We find that when determining CRC per sleep stage, differences between the two groups are further enhanced as compared with the main analysis (CRPS in N3 in Fig. 3; Fig. 8). Furthermore, this stratification also reveals changes that are not apparent when comparing CRC based on the full recordings (Fig. 6).

There are three physiological differences between pregnant and nonpregnant women which may contribute to the differences we see in CRC. The first is a difference in autonomic regulation; healthy pregnancy is an autonomic state characterized by increased sympathetic and decreased parasympathetic activity when compared with nonpregnant women of similar age (1, 11, 52). This is also apparent from our results, as the reduced amplitude and slope of the PRSA waveforms of the tachogram for the pregnant group (Fig. 5) can be explained by reduced parasympathetic activity (21, 44). This is further confirmed by the fact that the features describing these waveforms (DC, AC, IDR, IAR, SDR, and SAR, Table 3) are significantly lower in pregnant women, with large effect sizes (*P* < 0.001, *d* > 1). We observed similar results in previous work done in our group (11, 53). This reduced parasympathetic activity likely contributes to the lower levels of CRPS seen in the pregnancy group (blue boxplots in Fig. 3) (19). Note that CRPS is only higher for pregnant women for the PLR of 5:1 (Fig. 3). This is probably because pregnant women have higher HRs and similar respiratory rates to nonpregnant women (11, 31); this can also be seen for our subject cohorts in Table 1, indicating that a PLR with a high number of R-peaks within one respiratory cycle would be more likely to occur during pregnancy. The fact that nonpregnant have lower HRs likely contributes to the fact that more CRPS is seen for PLRs of 3:1 and 4:1; however, the total CRPS is also higher in nonpregnant women (Fig. 3).

Researchers have found that the occurrence of CRPS increases as parasympathetic activity increases (19). This is most prominent during N3 sleep, where parasympathetic activity is the highest and sympathetic activity is the lowest. During N3 sleep, HR decreases and baroreceptors, which are stretch receptors in the aortic arch and carotid sinuses that help regulate blood pressure, become more sensitive. This further promotes a state of regular respiration and gas exchange, triggering higher levels of CRPS (54). Tracking the statistically significant changes (*P* < 0.001) across sleep stages for the nonpregnant group (green boxplots in Fig. 4) illustrates this phenomenon well, as CRPS increases progressively from N1 to N3, with a difference of 15% from N1 to N3. Furthermore, consider the coupling from respiration to cardiac activity, which is represented in Fig. 8, where we see that the response in the BPRSA waveform is larger during N2 (Fig. 8*C*) than during REM (Fig. 8*D*), indicating reduced coupling under sympathetic dominant states such as REM.

This relationship between sleep architecture and CRPS is also described in the literature (19, 40). Assessing CRPS in older subjects, who, similar to pregnant women, have higher sympathetic and lower parasympathetic activity, reveals that while the prevalence of CRPS decreases with age, the relationship between CRPS and sleep architecture remains intact (19). We find that while this relationship is also present in healthy pregnant women, it is less prominent in this group when compared with the nonpregnant group (Fig. 4).

The second physiological difference which may impact maternal CRC results from the anatomical changes that occur in the maternal respiratory system during pregnancy (55). In a nonpregnant person, breathing is facilitated by the diaphragm and intercostal muscles, i.e., the muscles between the ribs. To inhale, the diaphragm flattens and the intercostal muscles contract to expand the ribcage, thereby creating a greater negative intrapleural pressure in the thoracic cavity and allowing air to fill the lungs. Thereafter, the diaphragm and intercostal muscles relax again, reducing the negative intrapleural pressure and forcing air out of the lungs (55). However, as the fetus grows during pregnancy, it pushes up against the diaphragm and in doing so reduces the ability of the diaphragm to descend; correspondingly, there is less expansion of the lungs in the inferior direction (anatomically speaking) (55). The maternal anatomy compensates for this by remodeling the ribcage to allow for more lateral expansion of the lungs during inspiration (55). Considering that the respiration in this study was measured with a thoracic band, this increased lateral expansion may result in the higher amplitude of the differences in the PRSA response in pregnant women (as compared with nonpregnant women) when APs are inhalations or exhalations (Figs. 7 and 8).

These changes in the thoracic cavity may also affect the blood flow to the heart, specifically, the venous return. The increase in negative intrapleural pressure during inspiration, along with the increased pressure that the descended diaphragm places on the abdominal cavity, increases the blood flow into the right atrium of the heart. This increased return to the right atrium and then ventricle results in increased stroke volume to the transpulmonary circulation (right ventricle pump). Thereafter, the increased preload to the left heart results in an increased stroke volume of the left ventricle. Correspondingly, HR also increases. This interaction facilitates respiratory sinus arrhythmia (RSA), a well-known coupling between the respiratory and cardiac systems, which is a measure of the amplitude of variation of the heartbeat intervals within respiratory cycles (19). The changes in the maternal respiratory system, in particular, the increased lateral and decreased inferior expansion of the lungs, could reduce RSA. This is likely the reason for the difference in the response seen in the BPRSA analysis of CRC from respiration to cardiac activity (Fig. 7). There is a larger response in the HR corresponding to inhalations and exhalations for nonpregnant women than for pregnant ones, which is confirmed by the large, statistically significant differences in MBA between the groups (*P* < 0.001, *d* > 1.2, Table 4). Furthermore, there is more regularity (confirmed by the significant differences in SampEn between the groups, *Stratification by sleep stages: coupling from respiration to cardiac activity*) in the BPRSA waveforms of nonpregnant women, suggesting that this aspect of their CRC is more regular and predictable than that of pregnant women.

Third, and lastly, there is increased physiological stress during pregnancy. Pregnancy is often referred to as a 9-mo stress test for the body (56, 57), and coupling between physiological systems reduces or even disappears under stress as the two subsystems start functioning more independently. Subsequently, the stress that pregnancy places on the body likely contributes to both the reduced CRPS and smaller BPRSA response seen in pregnant women. However, it is important to note here that while coupling reduces during pregnancy, it still occurs. Therefore, it seems that the mother’s body adapts well enough to the increased demands of healthy pregnancy, including the physiological stress, altered autonomic state, and remodeled respiratory system, to still maintain CRC.

CRC is a complex physiological phenomenon that is not yet fully understood in healthy individuals (58), much less in pregnant women. The literature concerning the latter is very limited. One research group found that CRC changes with gestational age (17). While we conversely found no relationship between CRC and progressing pregnancy (results), we should note that the gestational age range of our group is substantially smaller than theirs, which may be obscuring changes. Maternal CRC warrants further investigation. Investigating CRC provides additional insights into the physiology of pregnancy. Moreover, assessing CRC may offer opportunities for the early detection of pregnancy complications, as CRC is autonomically regulated and pregnancy complications are linked to autonomic dysfunction. The case for such assessments has already been made, as two investigations have demonstrated differences in CRC between healthy pregnant women and those with preeclampsia (15, 16).

When assessing CRC through the filter of a specific sleep stage, we find that not only are certain differences between pregnant and nonpregnant women greater than when assessed across the entire night recording (Figs. 4 and 8), but this stratification also reveals changes that are not apparent when using the entire night’s recording (Fig. 6). Therefore, we postulate that differences between healthy and complicated pregnancies might be further illuminated by comparing CRC per sleep stage, rather than based on the recordings of an entire night. It should be remembered, however, that stratifying by sleep stages potentially eliminates the effect of short sleep stages (less than 1 min in the case of the synchrogram and less than 3 min for BPRSA) as well as the impact of the transitions between the sleep stages. Further investigation is needed to understand the relationship between sleep stages transition and CRC.

A limitation of this work is that the measurement setup for the two groups differed; data from nonpregnant women were collected in a sleep laboratory, whereas those of pregnant women were collected at home. Stratifying the analyses by sleep stages aids in reducing the impact of these differing setups. However, it is not possible to reliably compare the Wake states between the groups. Furthermore, there is a small difference in age between the two groups (Table 1). As retrospective data are used from relatively small datasets (less than 60 participants each), it was not possible to age-match the two groups. This difference is likely too small to account for the differences seen between the pregnant and nonpregnant women. In addition, we did not find a relationship between age and the CRC indices. However, a prospective, dedicated, and age-matched study is needed to confirm our results. Furthermore, as expected from the literature (31), respiratory rates are comparable between the two groups but there is a difference in mean HR (Table 1). The relationship between mean HR and CRC has to our knowledge not been directly investigated, and future research in this area would add value to the field of CRC.

To conclude, this work offers novel insights into the physiology of pregnancy. We show that while CRC is present in healthy pregnancies, it occurs less often than in nonpregnant women. The sensitivity of CRC to pregnancy suggests that it might be an additional tool for assessing maternal health. In addition, assessing CRC per sleep stage will likely offer more meaningful information than assessing CRC across the entire night.

## DATA AVAILABILITY

Source data for this study are not publicly available due to privacy and ethical restrictions. The source data are available to verified researchers upon request by contacting the corresponding author.

## DISCLOSURES

R.V. is a cofounder and shareholder in Nemo Healthcare. P.F. and R.J. were employed by Philips Research at the time of this research. None of the other authors has any conflicts of interest, financial or otherwise, to disclose.

## AUTHOR CONTRIBUTIONS

M.B. and R.J. conceived and designed research; M.B., G.P., and P.F. analyzed data; M.B. interpreted results of experiments; M.B. prepared figures; M.B. drafted manuscript; M.B., G.P., P.F., M.M.v.G., M.M., J.O.v.L., R.V., and R.J. edited and revised manuscript; M.B., G.P., P.F., M.M.v.G., M.M., J.O.v.L., R.V. and R.J. approved final version of manuscript.
